# Impact of Music Therapy on Gait After Stroke

**DOI:** 10.7759/cureus.18441

**Published:** 2021-10-02

**Authors:** Anjali Daniel, Helene Koumans, Latha Ganti

**Affiliations:** 1 Emergency Medicine, Trinity Preparatory School, Winter Park, USA; 2 Emergency Medicine, Brown University, Providence, USA; 3 Emergency Medicine, Envision Physician Services, Plantation, USA; 4 Emergency Medicine, University of Central Florida College of Medicine, Orlando, USA; 5 Emergency Medicine, Ocala Regional Medical Center, Ocala, USA; 6 Emergency Medicine, HCA Healthcare Graduate Medical Education Consortium Emergency Medicine Residency Program of Greater Orlando, Olrando, USA

**Keywords:** emergency medicine, music-based therapies, musical motor feedback, music-supported therapy, rhythmic auditory stimulation, stroke rehabilitation, gait, hemorrhagic stroke, ischemic stroke, post stroke recovery

## Abstract

Music therapy or music-supported therapy is a therapeutic modality sometimes used during the rehabilitation phase after an acute ischemic or hemorrhagic stroke. The intervention suggests that the resulting audio-motor coupling can enhance motor function. Multiple clinical studies have reported various improvements-including cognitive, mood, and limb function. Gait impairment after stroke confers significant morbidity. The authors present a systematic review of randomized controlled trials that have examined the impact of music therapy on patient recovery, specifically on gait and ambulation.

## Introduction and background

Stroke remains the number one cause of long-term disability worldwide. Of the 15 million patients annually affected by stroke worldwide, the World Health Organization reports nearly a third of these patients die, and another third experienced permanent disability [1]. Stroke reduces mobility in more than one-half of stroke survivors ages 65 and above [2].

Mobility is diminished due to the sequelae that occur secondary to the infarcted brain. Motor deficits lead to a loss of voluntary muscle control, which ultimately leads to a restriction in activity. This results in paresis, spasticity, or disordered muscle contraction, and eventually muscle atrophy with or without contractures. For example, leg weakness gives rise to ambulatory difficulties, in turn increasing the likelihood of falls. Sensory deficits can lead to injuries due to a lack of awareness. A lack of position, sense, and movement is seen with proprioceptive deficits. The loss of independence observed among stroke patients can lead to depression and results in decreased cognitive ability. The aforementioned disabilities further isolate the stroke patient and reduce their participation in society as well as their motivation to recover [3].

The acute care of the stroke patient is relatively well organized, with aggressive time targets for administering thrombolytics, assessing for large vessel occlusion, and expediting bundled stroke care [4,5]. Many pharmacologic therapies for the acute post-stroke phase have been studied as well, including antibiotics, anticoagulants, and glycoprotein IIb/IIIa inhibitors [6-8]. After the acute phase post-hospital discharge, the remaining treatment consists primarily of rehabilitation.

Many types of rehabilitation strategies have been implemented and studied such as occupational, physical, and recreational therapy. This review focuses specifically on music therapy, and the purpose of this review is to summarize the impact of music-supported therapy (MST) on outcomes after stroke.

MST is a relatively new approach in treating disabilities rooted in neurologic dysfunction, such as stroke. MST is a non-invasive technique and does not require the administration of medications unless warranted by the study. This therapy focuses on a variety of goals, such as mitigating pain, aiding in a more rapid recovery, and improving quality of life and emotional status resulting from social interactions and music-related activities. The intervention also attempts to target improvements in cognitive function and physical rehabilitation. Music therapy uses the relationship between the patient and a credentialed professional to attain the varied goals of the individual using a wide range of approaches depending on the needs of the patient. Problems involving the upper limbs after stroke have been shown to have significant improvements in range of motion and functional mobility resulting from MST intervention [9]. As compared to the control groups in many of the studies conducted, many experimental groups participating in MST also exhibited increased improvements in blood flow and joint flexion required for a more rapid healing process and general recovery [10]. MST has also had a tremendous impact on recovering stroke patients with regard to their emotional and psychological status. Through the varied activities involved in MST interventions, such as passive listening to music, patients have reported a significant positive change in their quality of life and perception of themselves [11]. MST interventions may also include rhythmic auditory stimulation (RAS), rhythmic- and music-based therapies (R-MT), musical motor feedback (MMF), and active listening, a technique in which the patients play an instrument or compose their own music. Active listening for stroke patients proves to have a significant improvement on their neurorehabilitation and cognitive processing, as well as their emotional state and social interactions [12]. With an enriched sound environment and daily listening to music, the prediction for the patient’s recovery time is also more accurate as they feel a better sense of control and the motivation to move forward in their recovery [13].

## Review

Methods

This systematic review has been reported according to the Preferred Reporting Items for Systematic Review (PRISMA) statement [14]. A protocol for this review was not registered prospectively.

Search Strategy

A systematic literature search was conducted in August 2021 of the following electronic databases: MEDLINE, PubMed, PubMed Central, and Google Scholar. Databases were searched using a combination of the following keywords: stroke OR ischemic stroke OR intracerebral hemorrhage OR hemorrhagic stroke AND music therapy OR MST. Additional articles were identified from reference lists of retrieved articles.

Eligibility Criteria

Studies were included if they met the following Population, Intervention, Controls, Outcome, Study design, Time (PICOS) criteria. The population had to include adults (aged 18 years and above) diagnosed with ischemic or hemorrhagic stroke, as confirmed by computer tomography or magnetic resonance imaging and by diagnostic guidelines updated by the American Heart Association/American Stroke Association. The interventions had to be sound-based, including music listening (MLI) or listening to rhythmic sequences (RAS). These could be performed by various instruments, such as a metronome or synthesizer. At least one group of participants had to perform a gait-related task in this condition. The control group had to include a similar motor act, which is to be performed without listening to music or rhythmic sequences. The outcome measures had to assess gait or ambulation. The study design only included randomized controlled trials. The time frame included studies published between January 2010 and August 2021. Non-human and studies in languages other than English were excluded from the review.

Study Selection and Data Abstraction

**Figure 1:**
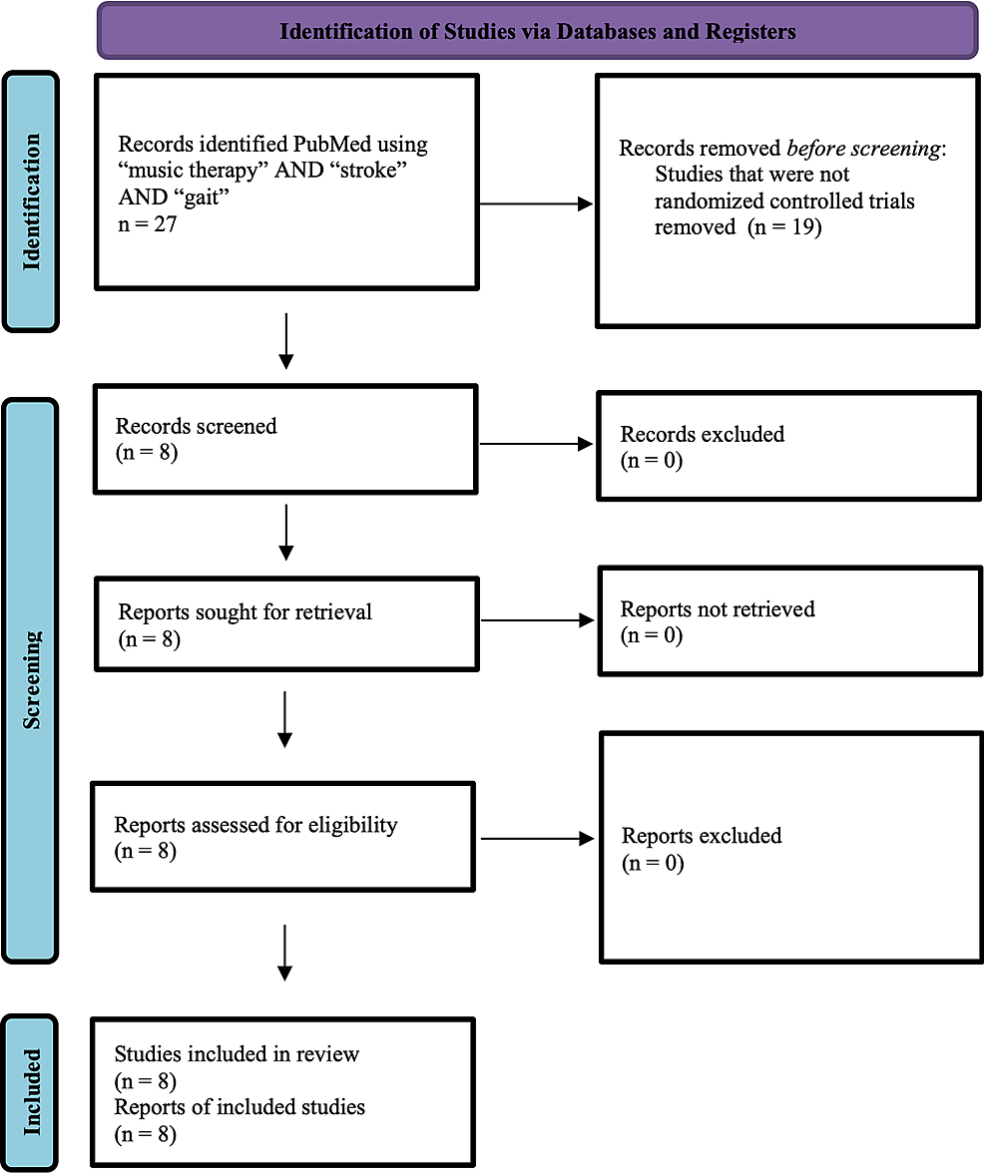
Identification of studies via databases and registers

All potentially relevant articles for this qualitative analysis were independently assessed for inclusion in the review by each reviewer. Data were extracted onto predesigned data abstraction forms. Data abstracted included the year of study, the number of participants in study and control groups, and outcomes. For studies in which only abstracts were available, the reviewers contacted the medical school librarian to determine whether a full article was available. Disagreements were resolved by consensus. Figure 1 describes the process of identification of studies.

Results

Using the search terms “music therapy or “music supported therapy” and “stroke” and “gait” retrieved 266 articles. When this search was limited to randomized controlled trials, a total of eight studies met the criteria. Table 1 summarizes the included studies.

**Table 1:** Summary of included studies RAS: rhythmic auditory simulation, MMF: musical motor feedback, R-MT: rhythmic- and music-based therapies, H-RT: horse-riding therapy, MIDI: musical instrument digital interface

Title	Year of study	First author	n	Description of MST	Duration of MT sessions	Duration of intervention
The value of exercise rehabilitation program accompanied by experiential music for recovery of cognitive and motor skills in stroke patients	2018	George Fotakopoulos [15]	65	Daily listening to experiential/traditional music	45 min/day, four training sessions per week	Six months
Intensive gait training with rhythmic auditory stimulation in individuals with chronic hemiparetic stroke: a pilot randomized controlled study	2014	Yuri Cha [16]	20	RAS	30 min/day, five days/week	Six weeks
Musical motor feedback (MMF) in walking hemiparetic stroke patients: randomized trials of gait improvement	2003	Michael Schauer [17]	23	Therapy sessions with MMF	20 min/day, five days/week (15 sessions)	Three weeks
The effect of rhythmic auditory stimulation (RAS) on physical therapy outcomes for patients in gait training following stroke: a feasibility study	2009	Rebecca Hayden [18]	15	RAS-enhanced gait training group 1: RAS to enhance 30 traditional physical therapy gait-training sessions group 2 (wait-list control A): 10 traditional physical therapy gait training sessions followed by 20 RAS enhanced gait training sessions group 3 (wait-list control B): 20 traditional physical therapy gait training sessions followed by 10 RAS enhanced gait training sessions	Minimum of one session daily for eight to ten mins	30 days
Long-term improvements after multimodal rehabilitation in late phase after stroke: a randomized controlled trial	2017	Lina Bunketorp-Käll [19]	41	Group 1: R-MT, group 2: H-RT, group 3 (control): received R-MT one year after inclusion	Two sessions/weeks	12 weeks
Effect of rhythm of music therapy on gait in patients with stroke	2021	Yao Wang [20]	60	“metronome was used to coordinate with the patient’s walking velocity” in first session; “music [with] familiar melody of patients, according to the walking velocity obtained from the first therapy, in order to help patients control their walking rhythm with the playing rhythm as the indicator signal. At the same time, patients were required to perform walking training according to the playing music rhythm” in second session; “the metronome was used again to measure the walking velocity, and this was used in the next walking training as the basic speed of the next music therapy”	60 min/session, three sessions/day	Four weeks
Effects of a music-based rhythmic auditory stimulation on gait and balance in subacute stroke	2021	Samira Gonzalez-Hoelling [21]	28	“15 min of general body warming following the rhythm with a metronome, a main part of the session with 60 min of music-based RAS exercises, and closure with 15 min of relaxation exercises” (Ronnie Gardiner Method)	Received music-based rhythmic auditory stimulation for 90 min, three times per week	40-60 days (depending on the length of hospital stay)
Effect of rhythmic auditory stimulation on gait and balance in hemiplegic stroke patients	2014	Jee Hyun Suh [22]	16	four-step gait training with RAS using a digital MIDI software, single tone series in 4/4 time signature	gait training with RAS for 15 minutes, five sessions/week	three weeks

Eight studies investigating music therapy in stroke rehabilitation were included in the review, of which four conducted RAS, one conducted MMF, one conducted R-MT, and two conducted more individually defined music interventions [15-22]. The majority of included studies - six of the eight - reported positive outcomes on gait measures among stroke patients who received music or rhythmic therapies [15-17,19,20,22]. The findings of the remaining two studies, conducted by Hayden et al. and Gonzalez-Hoelling et al., were unclear; improvements were observed in both the study groups and the control groups with no evident distinctions [18,21]. Both Hayden et al. and Gonzalez-Hoelling et al. acknowledged the clinical feasibility of music-based therapies and recommend further study to investigate the effectiveness of such therapies on gait. Both of the aforementioned studies conducted RAS interventions, and although improvements were observed in patients’ one-limb stance, cadence, velocity, stride length, and posture head tilt as well as their Functional Ambulation Category, statistically significant differences were not evident with the respective control groups [18,21].

The studies conducted by Fotakopoulos and Kotlia, Cha et al., Schauer and Mauritz, Bunketorp-Käll et al., Wang et al., and Suh et al. reported positive results on gait among stroke patients receiving music therapy [15-17,19,20,22]. Not only were gait velocity and results on balance and ambulation assessments improved among patients in the respective intervention groups, but also long-term and quality of life improvements were observed. Fotakopoulos and Kotlia, whose intervention included daily listening to experiential and traditional music, found recovery was higher and mood profile was improved when treatment involved the music-based exercise program [15]. Gait training with RAS, as per Cha et al., yielded considerable improvements on patients’ “gait velocity, cadence, stride length on the affected side, and double support period on the affected side” [16]. Schauer and Mauritz detailed the improvements in average gait velocity and stride execution among patients in the MMF intervention groups, though they noted gait cadence was unaffected [17]. According to Bunketorp-Käll et al., sustained “meaningful recovery” was observed among patients who conducted R-MT received higher scores on gait and balance assessments as compared to the control group [19]. Stroke patients who received music therapy exhibited increased stride length, cadence, and maximum velocity as well as improved scores on motor functioning and balance assessments [20]. Another clinical trial conducted showed dramatic improvements in gait velocity, stride length, cadence, overall stability index, and mediolateral index in the RAS group as compared to the control group [22].

Discussion

The purpose of this systematic review was to evaluate the effectiveness of MST on gait improvement and recovery of cognitive and motor skills in stroke patients. The results show that MST can be beneficial in improving gait and ambulation as well as enhanced cognitive and motor function in the life of stroke patients [11]. Gait involves the repetitive pattern involving the steps and strides an individual take [16]. Cadence is the total number of times one’s feet hit the ground in a one-minute period. An average walking cadence for adults aged 60-69 years is 65 steps/min [23]. For post-stroke patients, the number of steps is diminished [24]. Decreased cadence can lead to decreased ambulation, leading to the general decline.

The subjects of the included studies exhibited symptoms as a result of subacute stroke, hemiplegic stroke, hemiparetic stroke, hemorrhagic stroke, and ischemic stroke. Through the systematic review, it was concluded that MST can improve the function of ambulation, cognitive function, and motor skills [4]. However, only six of the eight studies reported clear results of a positive outcome on gait ability from direct application of MST [15-17,19,20,22]. The cases conducted by Hayden and Gonzalez-Hoelling et al. both reported findings that were unclear and showed no observable distinctions between experimental and control groups [18,21]. The MST interventions implemented to reveal positive outcomes included daily listening, MMF, RAS, and therapy sessions involving physical movement with music in the background or as direct usage [15-17,19,20,22]. RAS is defined as an application of pulsed rhythms or stimulation through music to improve gait-related movements [25]. When patients trained with the rhythm of the music, muscle contractions, balance, and functional improvements were observed.

## Conclusions

MST has the ability to create significant improvement in the lives of recovering stroke patients. The use of MST can yield positive outcomes and sustained recovery for stroke patients and is clinically feasible. Rhythmic stimulation can synchronize movement and increase the flexion of the joints needed to recover. Daily listening to music improves the emotional state of the patient, enabling them to feel an increased motivation to recover and a more positive quality of life.

MST and its diverse methods of implementation are noninvasive, relatively inexpensive, and effective interventions in improving stroke patient recovery and overall function. It is recommended that these clinical treatments be further studied in larger, more diverse patient populations and considered in routine clinical practice and treatment. MST can confer significant improvements in gait function and ambulation in stroke patients. Given its clinical feasibility, MST - including RAS, R-MT, and MMF - is worth continuing to study as a valuable component in post-stroke recovery and rehabilitation.
